# Supplementary figures and images for: Prolonged cell cycle arrest in response to DNA damage in yeast requires the maintenance of DNA damage signaling and the spindle assembly checkpoint
Source: eLife. 2024 Dec 10;13:RP94334. doi: 10.7554/eLife.94334 (PMC11630823; doi:10.7554/eLife.94334)

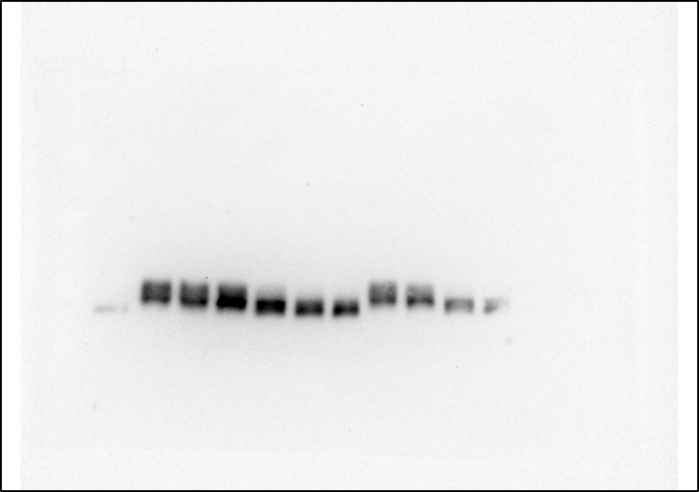

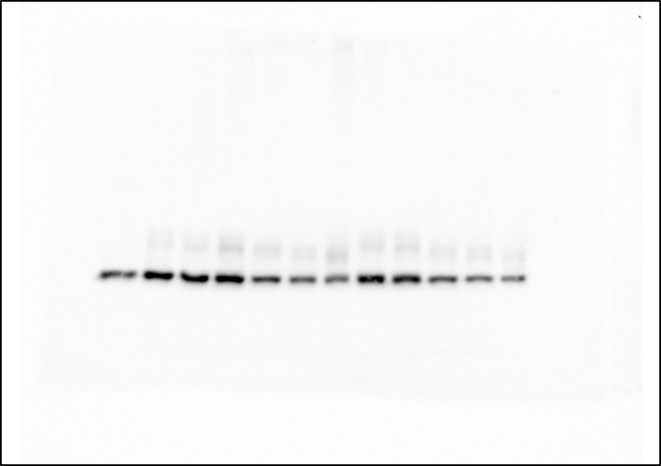


Figure 1 – Source Data 1. Original membranes corresponding to Figure 1, panel C.

Supplement: Figure 1—source data 1. [file elife-94334-fig1-data1.zip › Figure 1 - Source Data 1/Figure 1 - Source Data 1.docx]

### Myc and Rad53 antibody

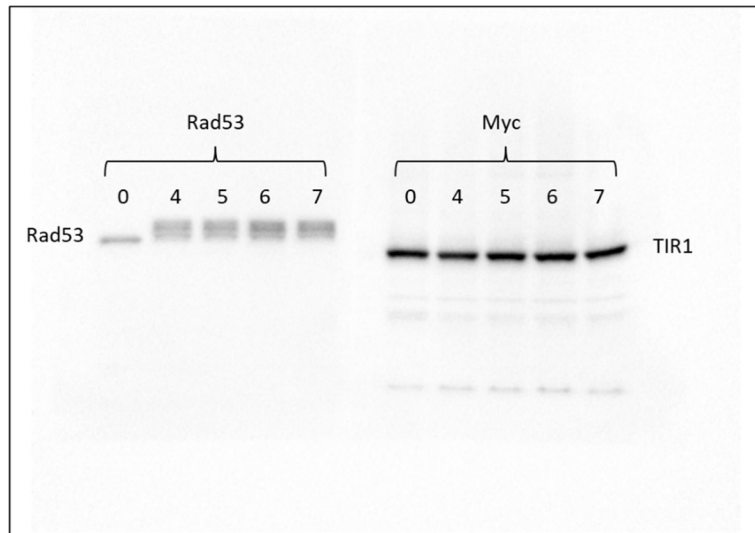

Figure 3 – Source Data 1. Original membranes corresponding to Figure 3, panel A.

Supplement: Figure 3—source data 1. [file elife-94334-fig3-data1.zip › Figure 3 - Source Data 1/Figure 3 - Source Data 1.pdf]

## Myc antibody

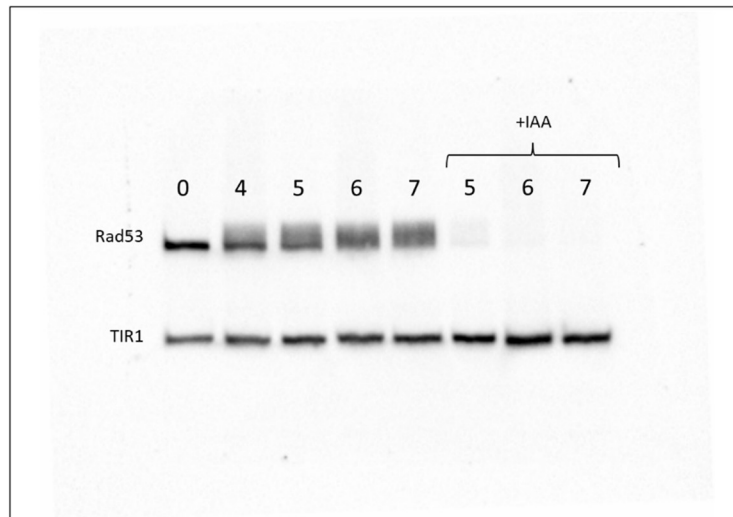

Figure 3 – Source Data 3. Original membranes corresponding to Figure 3, panel C.

Supplement: Figure 3—source data 3. [file elife-94334-fig3-data3.zip › Figure 3 - Source Data 3/Figure 3 - Source Data 3.pdf]

**Myc and Pgk1 antibody**

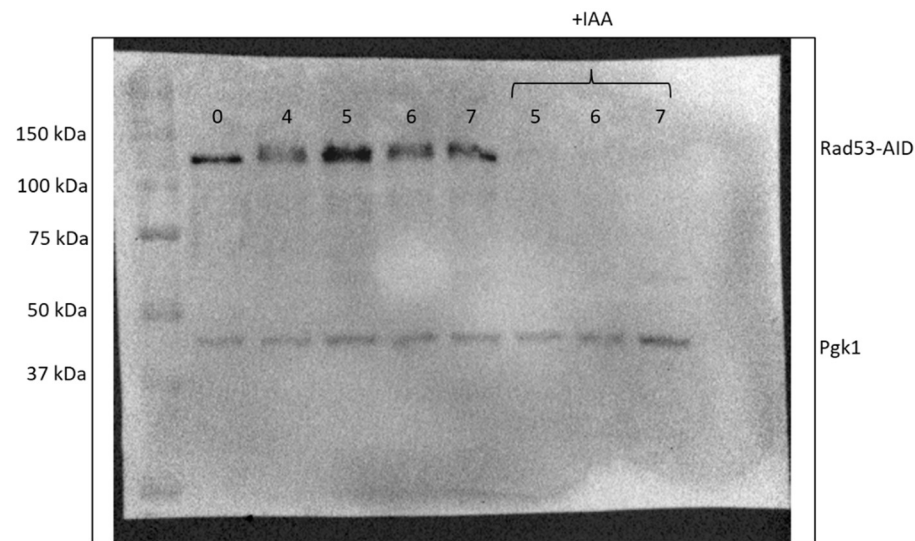

Figure 5 – Source Data 3. Original membranes corresponding to Figure 3, panel C.

Supplement: Figure 5—source data 3. [file elife-94334-fig5-data3.zip › Figure 5 - Source Data 3/Figure 5 - Source Data 3.pdf]

**Myc and Pgk1 antibody**

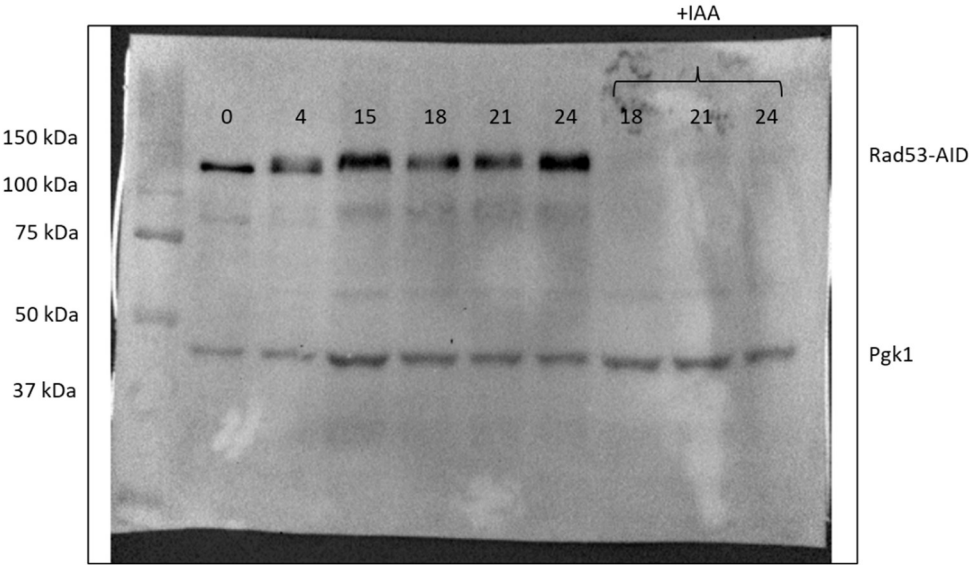

Figure 5 – Source Data 5. Original membranes corresponding to Figure 3, panel D.

Supplement: Figure 5—source data 5. [file elife-94334-fig5-data5.zip › Figure 5 - Source Data 5/Figure 5 - Source Data 5.pdf]
