# Supplementary material for: Prolonged cell cycle arrest in response to DNA damage in yeast requires the maintenance of DNA damage signaling and the spindle assembly checkpoint
Source: eLife. 2024 Dec 10;13:RP94334. doi: 10.7554/eLife.94334 (PMC11630823; doi:10.7554/eLife.94334)
Supplement: Figure 1—source data 3. [file elife-94334-fig1-data3.zip › Figure 1 - Source Data 3/Figure 1 - Source Data 3.pdf]

Myc antibody

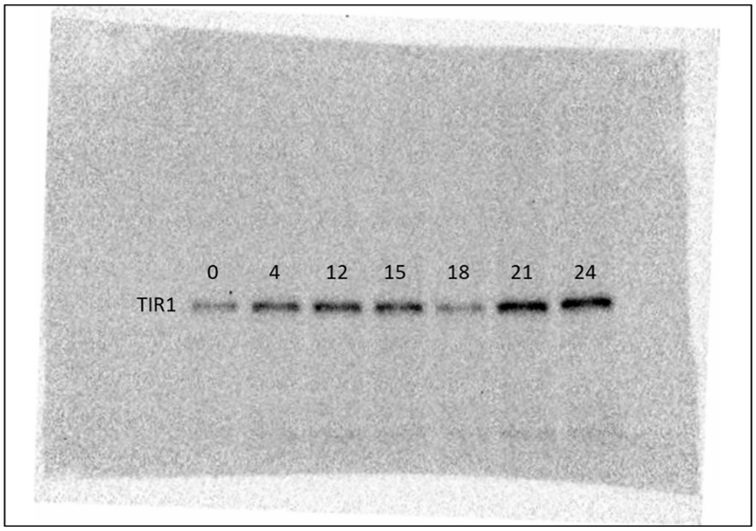

Rad53 antibody

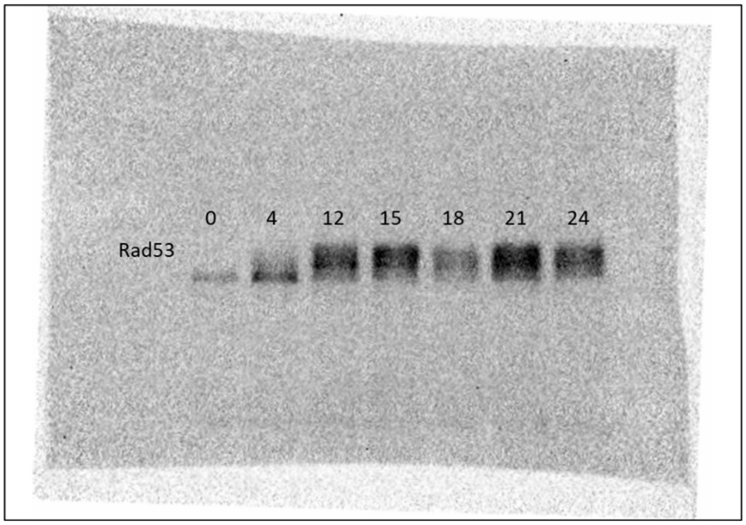

Figure 1 – Source Data 3. Original membranes corresponding to Figure 1, panel F.
