# Supplementary material for: Prolonged cell cycle arrest in response to DNA damage in yeast requires the maintenance of DNA damage signaling and the spindle assembly checkpoint
Source: eLife. 2024 Dec 10;13:RP94334. doi: 10.7554/eLife.94334 (PMC11630823; doi:10.7554/eLife.94334)
Supplement: Figure 2—source data 1. — The 7 hr ± IAA samples were added to the last two wells. [file elife-94334-fig2-data1.zip › Figure 2 - Source Data 1/Figure 2 - Source Data 1.docx]

**Myc blot**

**
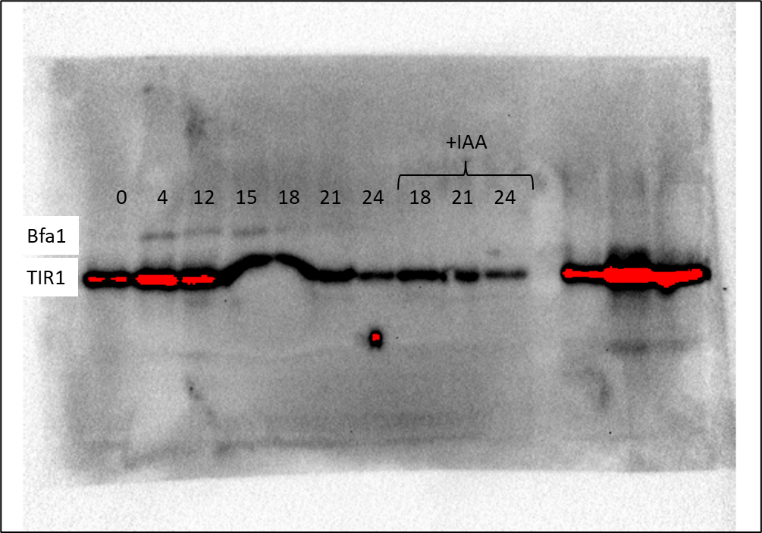
**

**Myc and Pgk1 blot**

**
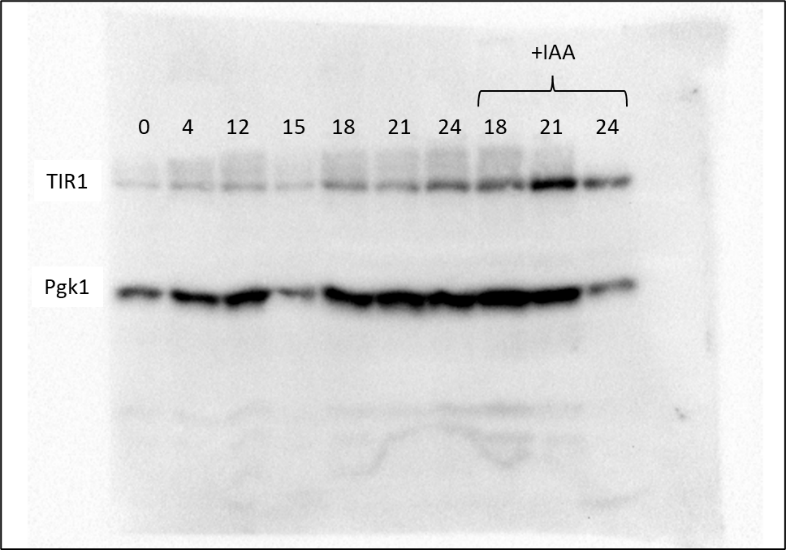
**

**Rad53 blot**

**
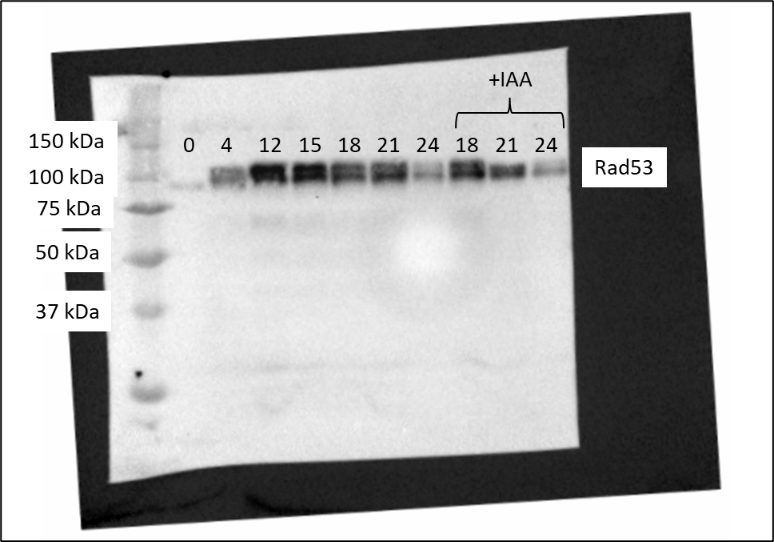
**

Figure 7 – Source Data 3. Original membranes corresponding to Figure 7 - panel C.
