# Supplementary material for: Prolonged cell cycle arrest in response to DNA damage in yeast requires the maintenance of DNA damage signaling and the spindle assembly checkpoint
Source: eLife. 2024 Dec 10;13:RP94334. doi: 10.7554/eLife.94334 (PMC11630823; doi:10.7554/eLife.94334)
Supplement: Figure 2—figure supplement 2—source data 1. [file elife-94334-fig2-figsupp2-data1.zip › Figure 2 - figure supplement 2 - Source Data 1/Figure 2 - figure supplement 2 -Source Data 1.pdf]

Rad53 antibody

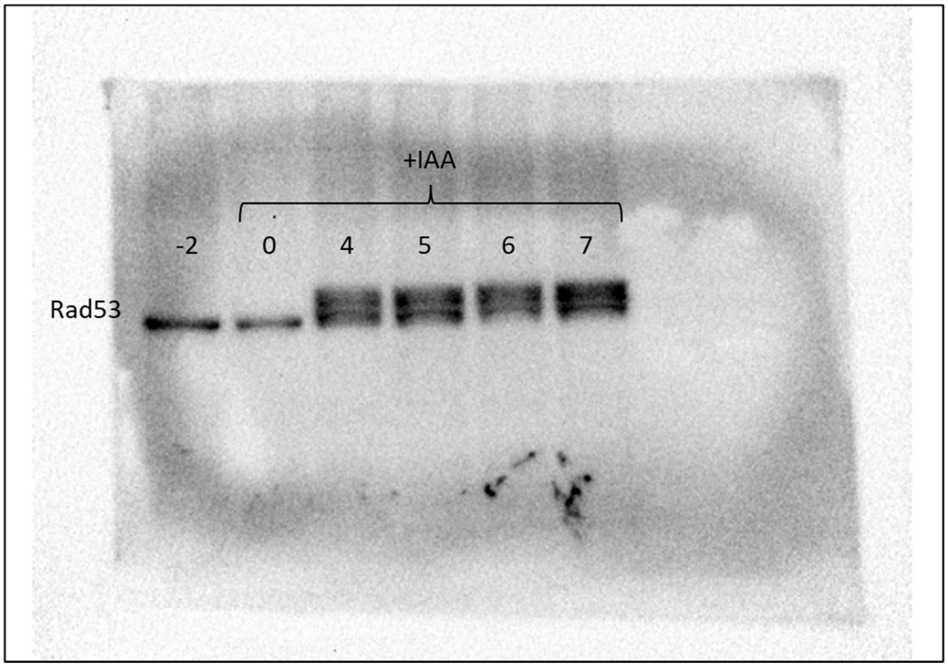

Figure 2 - figure supplement 2 – Source Data 1. Original membranes corresponding to Figure 2 - figure supplement 2, panel A.
