# Supplementary material for: Prolonged cell cycle arrest in response to DNA damage in yeast requires the maintenance of DNA damage signaling and the spindle assembly checkpoint
Source: eLife. 2024 Dec 10;13:RP94334. doi: 10.7554/eLife.94334 (PMC11630823; doi:10.7554/eLife.94334)
Supplement: Figure 3—source data 5. [file elife-94334-fig3-data5.zip › Figure 3 - Source Data 5/Figure 3 - Source Data 5.pdf]

Myc antibody

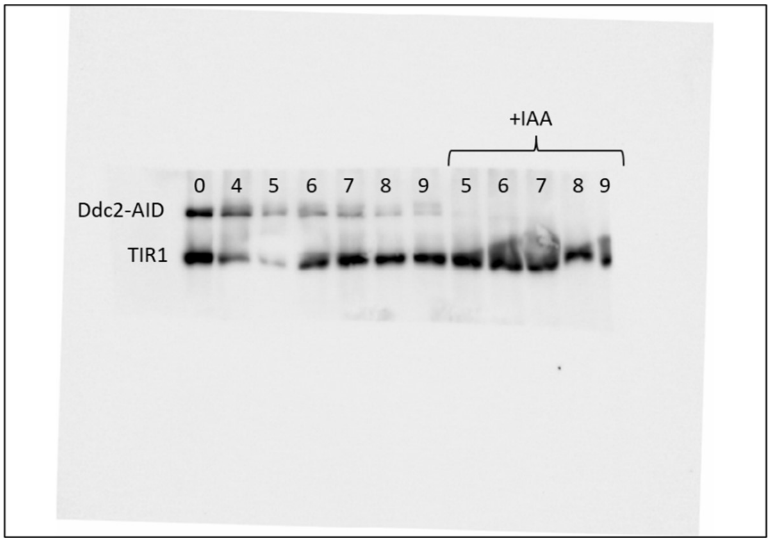

Rad53 antibody

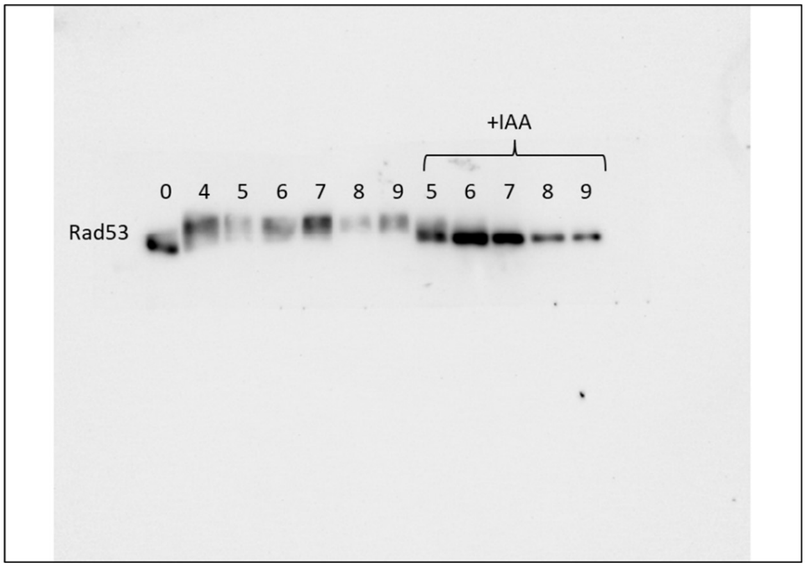

Figure 3 – Source Data 5. Original membranes corresponding to Figure 3, panel D.
