# Supplementary material for: Prolonged cell cycle arrest in response to DNA damage in yeast requires the maintenance of DNA damage signaling and the spindle assembly checkpoint
Source: eLife. 2024 Dec 10;13:RP94334. doi: 10.7554/eLife.94334 (PMC11630823; doi:10.7554/eLife.94334)
Supplement: Figure 6—figure supplement 3—source data 1. [file elife-94334-fig6-figsupp3-data1.zip › Figure 6 - figure supplement 3 - Source Data 1/Figure 6 - figure supplement 3 -Source Data 1.pdf]

Myc blot

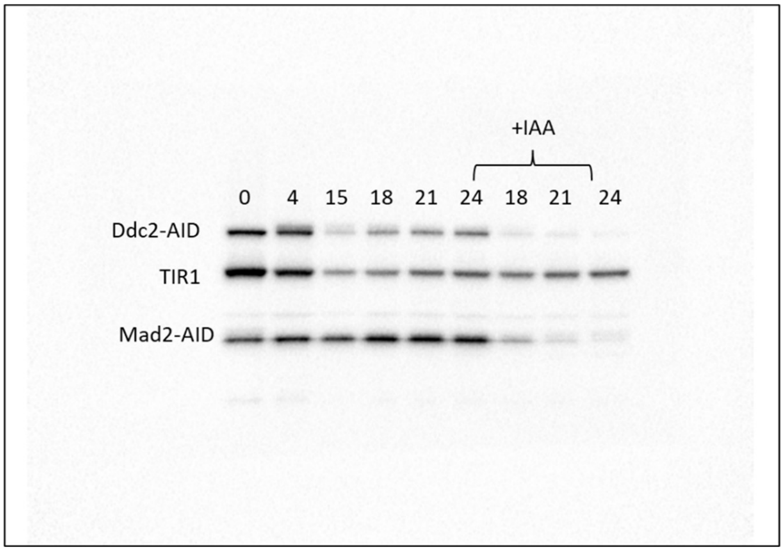

Rad53 blot

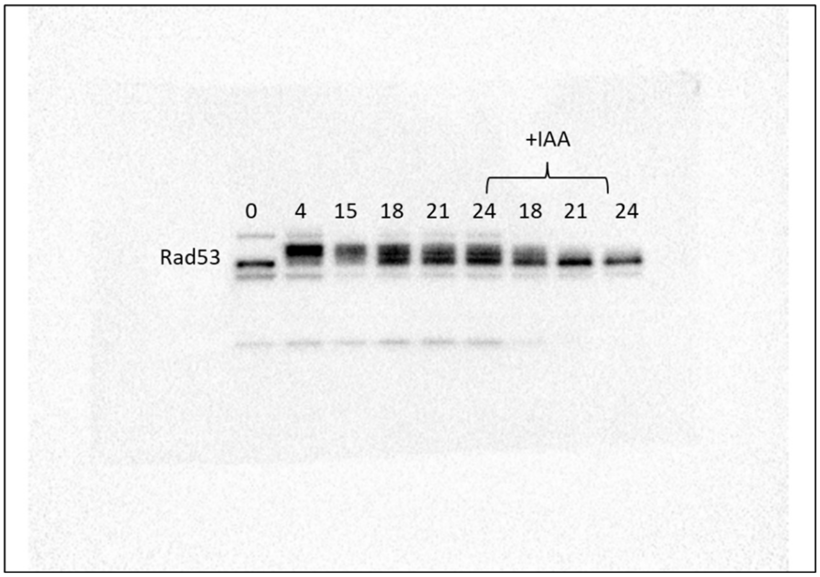

Figure 6 - figure supplement 3 – Source Data 1. Original membranes corresponding to Figure 6 - figure supplement 3, panel A.
