# Supplementary material for: Prolonged cell cycle arrest in response to DNA damage in yeast requires the maintenance of DNA damage signaling and the spindle assembly checkpoint
Source: eLife. 2024 Dec 10;13:RP94334. doi: 10.7554/eLife.94334 (PMC11630823; doi:10.7554/eLife.94334)
Supplement: Figure 7—figure supplement 1—source data 1. [file elife-94334-fig7-figsupp1-data1.zip › Figure 7 - figure supplement 1 - Source Data 1/Figure 7 - figure supplement 1 -Source Data 1.pdf]

Myc and Pgk1 blot

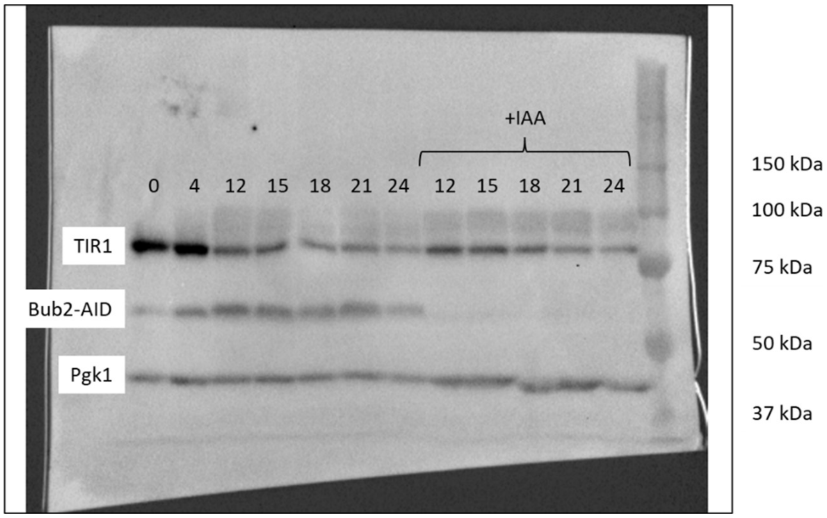

Rad53 blot

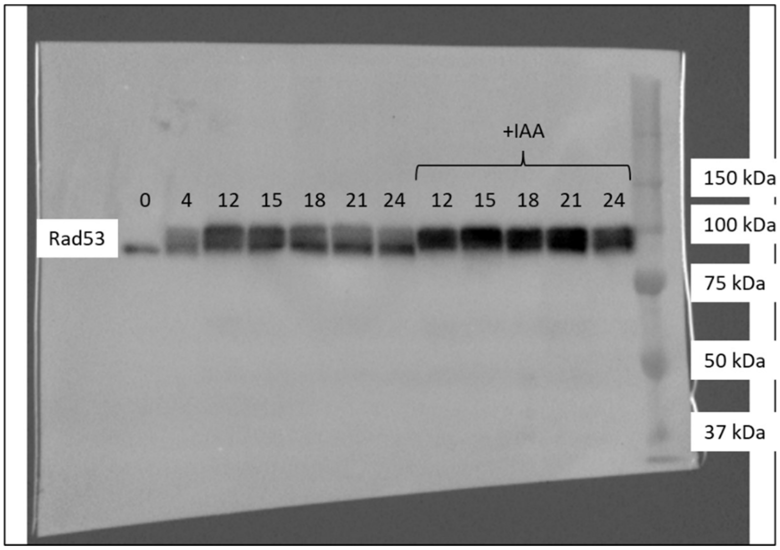

Figure 7 - figure supplement 1 – Source Data 1. Original membranes corresponding to Figure 7 - figure supplement 1, panel A.
