# Supplementary material for: Prolonged cell cycle arrest in response to DNA damage in yeast requires the maintenance of DNA damage signaling and the spindle assembly checkpoint
Source: eLife. 2024 Dec 10;13:RP94334. doi: 10.7554/eLife.94334 (PMC11630823; doi:10.7554/eLife.94334)
Supplement: Supplementary file 1. — (a) Strains used in this study. (b) Primers used in this study. [file elife-94334-supp1.docx]

**Supplementary file 1a – Strains**

| **Strain** | **Parent** | **Genotype** | **Reference** |
| --- | --- | --- | --- |
| JKM179 |  | *MATα ade1 leu2-3 lys5 trp1::hisG ura3-52 hoΔ hmlΔ::ADE1 hmrΔ::ADE1 ade3::GAL::HO* | Lee et al. Cell 1998 |
| DW184 | JKM179 | *TIR1-myc6::URA3* | This study |
| DW417 | JKM179 | *HOcse6::HPH TIR1-myc6::URA3* | This study |
| DW418 | DW417 | *Ddc2-AID*-9xMyc::KAN* | This study |
| DW419 | DW417 | *Rad9-AID*-9xMyc::KAN* | This study |
| DW420 | DW417 | *Rad24-AID*-9xMyc::KAN* | This study |
| DW421 | DW417 | *Rad53-AID*-9xMyc::KAN* | This study |
| DW426 | DW417 | *chk1∆::NAT* | This study |
| DW647 | DW418 | *Ddc2-AID*-9xMyc::KAN chk1∆::NAT* | This study |
| DW427 | DW419 | *Rad9-AID*-9xMyc::KAN chk1∆::NAT* | This study |
| DW428 | DW420 | *Rad24-AID*-9xMyc::KAN chk1∆::NAT* | This study |
| DW429 | DW421 | *Rad53-AID*-9xMyc::KAN chk1∆::NAT* | This study |
| DW625 | DW417 | *dun1∆::KAN* | This study |
| DW626 | DW417 | *Dun1-AID*-9xMyc::KAN* | This study |
| DW641 | DW626 | *Dun1-AID*-9xMyc::KAN chk1∆::NAT* | This study |
| FZ009 | DW417 | *Mad2*-9xMyc-AID::NAT* | This study |
| FZ010 | DW418 | *Ddc2-AID*-9xMyc::KAN Mad2*-9xMyc-AID::NAT* | This study |
| DW455 | DW417 | *mad2Δ::KAN* | This study |
| GM180 | JKM179 | *pGal::Ddc2::LEU2* | (Memisoglu et al. 2019) |
| DW648 | GM180 | *HOcse6::HPH pGal::Ddc2::LEU2* | This study |
| DW649 | DW648 | *HOcse6::HPH pGal::Ddc2::LEU2 mad2∆::NAT* | This study |
| DW642 | JKM179 | *HOcse6::HPH Ddc2-AID*-9xMyc::KAN* | This study **a** |
| DW643 | JKM179 | *HOcse6::HPH Rad9-AID*-9xMyc::KAN* | This study **b** |
| DW644 | JKM179 | *HOcse6::HPH Rad24-AID*-9xMyc::KAN* | This study |
| DW645 | JKM179 | *HOcse6::HPH Rad53-AID*-9xMyc::KAN* | This study |
| DW650 | GM180 | *pGal::Ddc2::LEU2 mad2∆::NAT* | This study |
| GM539 | JKM179 | *Ddc2-9xMyc::KAN* | (Memisoglu et al. 2019) |
| FZ001 | JKM179 | *MATα HOcse6::HPH* | This study |
| JY542 | JKM179 | *HOcse6::HPH tel1∆::KAN* | This study |
| FZ024 | FZ001 | *HOcse6::HPH Rad9-AID*-9xMyc::KAN* | This study |
| FZ025 | FZ001 | *HOcse6::HPH Rad24-AID*-9xMyc::KAN* | This study |
| FZ026 | FZ001 | *HOcse6::HPH Rad53-AID*-9xMyc::KAN* | This study |
| FZ173 | FZ025 | *HOcse6::HPH Rad24-AID*-9xMyc::KAN TIR1(F74G)::URA3* | This study |
| FZ174 | FZ024 | *HOcse6::HPH Rad9-AID*-9xMyc::KAN TIR1(F74G)::URA3* | This study |
| FZ175 | FZ026 | *HOcse6::HPH Rad53-AID*-9xMyc::KAN TIR1(F74G)::URA3* | This study |
| YSL53 | JKM179 | *HOcse5::URA3* | (Lee et al. 1998) **c** |
| GEM188 | JKM179 | *HOcse2::LYS2* | This study |
| FZ201 | DW419 | *Rad9-AID*-9xMyc::KAN pRAD9-AID*-9xMyc* | This study |
| FZ155 | DW417 | *bfa1∆::KAN* | This study |
| yMA11 | JKM179 | H2A-S129A H2B-T129A | This study |
| yMA12 | JKM179 | H2AS129E H2B-T129E | This study |
| yMA13 | JKM179 | H2B-T129A | This study |
| yMA14 | JKM179 | H2B-T129E | This study |
| yBL257 | JKM179 | H2A-S129E | This study |
| yBL259 | JKM179 | H2A-S129A | This study |
| FZ062 | DW417 | *Mad1*-9xMyc-AID::NAT* | This study |
| FZ165 | DW417 | *Bfa1*-9xMyc-AID::NAT* | This study |
| FZ167 | DW417 | *Bub2*-9xMyc-AID::NAT* | This study |

1. *HOcse6::HPH* is the an HO-cut site 42 kb away from the centromere on chromosome VI.
2. *HOcse5::URA3* is the an HO-cut site 36 kb away from the centromere on chromosome V.
3. *HOcse2::LYS2* is the an HO-cut site 230 kb away from the centromere on chromosome II.

**Supplementary file 1b – Primers**

| **Oligo** | **Sequence** | **Use** |
| --- | --- | --- |
| GAT1p1B | GCTCAGTGTGCGTTATGCTT | Primer to add the second HO-cut site on chromosome VI |
| GAT1p2B | TTCAGGTCTCGGTTGCTCTT | Primer to add the second HO-cut site on chromosome VI |
| VE162 Ddc2-AID For | ATCTAACCACACTAGAGGAGGCCGATTCATTATATATCTCAATGGGACTGCCTAAAGATCCAGCCAAACCTCC | C-terminal AID tag for Ddc2 (forward) |
| VE163 Ddc2-AID Rev | ATTACAAGGTTTCTATAAAGCGTTGACATTTTCCCCTTTTGATTGTTGCCCAGTATAGCGACCAGCATTCACATAC | C-terminal AID tag for Ddc2 (reverse) |
| DW217 Rad9-AID 1F | GGTTTTCACGATGATATTACGGACAATGATATATACAACACTATTTCTGAGGTTAGACCTAAAGATCCAGCCAAACCTCC | C-terminal AID tag for Rad9 (forward) |
| DW218 Rad9-AID 1R | CTAAATTTTTTTTTATTTAATCGTCCCTTTCTATCAATTATGAGTTTATATATTTTTATAATTCAGTATAGCGACCAGCATTCACATAC | C-terminal AID tag for Rad9 (reverse) |
| DW208 Rad24-AID 1F | CAGATTCAGATCTGGAAATACTCCCTAAAGATCCAGCCAAACCTCC | C-terminal AID tag for Rad24 (forward) |
| DW209 Rad24-AID 1R | GTGGAATATTTCCTGGGGTTTTCTCGTCAAATTTAAAGAGTAAAAAGCCTAAAGATCCAGCCAAACCTCC | C-terminal AID tag for Rad24 (reverse) |
| DW199 Rad53AID 1F | GGTTAAAAGGGCAAAATTGGACCAAACCTCAAAAGGCCCCGAGAATTTGCAATTTTCGCCTAAAGATCCAGCCAAACCTCC | C-terminal AID tag for Rad53 (forward) |
| DW200 Rad53AID 1R | CCATCTTCTCTCTTAAAAAGGGGCAGCATTTTCTATGGGTATTTGTCCTTGGCAGTATAGCGACCAGCATTCACATAC | C-terminal AID tag for Rad53 (reverse) |
| dw418 Dun1 1F | CGAGAGTAACAAGTAAAGGGGCTTAACATACAGTAAAAAAGGCAATTATAGTGAAGATGCCTTGACAGTCTTGACGTGC | Genomic deletion of Dun1 (forward) |
| dw419 dun1 1R | GATACTTGGAAAAATCCAGATTCAAACAATGTTTTTGAAATAATGCTTCTCATGTTTACGCACTTAACTTCGCATCTG | Genomic deletion of Dun1 (reverse) |
| Sp01Dun1aidF | CAATAAAATACCCAAAACATACTCAGAATTATCTTGCCTCCCTAAAGATCCAGCCAAACCTCC | C-terminal AID tag for Dun1 (forward) |
| SP02Dun1aidR | CCAGATTCAAACAATGTTTTTGAAATAATGCTTCTCATGTCAGTATAGCGACCAGCATTCACATAC | C-terminal AID tag for Dun1 (reverse) |
| BL189-Chk1 1F | TCAGCCACTGGTCATCCCGT | Genomic deletion Chk1 (forward) |
| BL190-Chk1 1R | GTTGGGGGGAGATGGTAACG | Genomic deletion Chk1 (reverse) |
| FZ013 Mad2-AID 1F | CATTCTCTACCAACGATCATAAAGTTGGTGCGCAGGTCAGCTATAAATATCCTAAAGATCCAGCCAAACCTCC | C-terminal AID tag for Mad2 (forward) |
| FZ014 Mad2-AID 1R | CGAGATTTTTTTGGACTTCCGTCTTTTTTTTTTTTTTTGACTTGAATTCTAGATATCATCGATGAATTCGAGCTCG | C-terminal AID tag for Mad2 (reverse) |
| FZ096 Mad1-AID 1F | GGCAACAATAACATTGCGTCTGTGGGAACAGCGACAAGCCAAA CCTAAAGATCCAGCCAAACCTCC | C-terminal AID tag for Mad1 (forward) |
| FZ097 Mad1-AID 1R | GGAGTTTATCATATTATAAAACCGATTACTATTATCTATTAGAAATGTATATACAC GATATCATCGATGAATTCGAGCTCG | C-terminal AID tag for Mad1 (reverse) |
| FZ147 Bub2-AID 1F | GACCACTTGACCGACCCAGACATATATATACCG CGTACGCTGCAGGTCGAC | C-terminal AID tag for Bub2 (forward) |
| FZ148 Bub2-AID 1R | CGTTGTAGAATTAAACGATAAAATATAATATTTCTTCACATAGT ATCGATGAATTCGAGCTCG | C-terminal AID tag for Bub2 (reverse) |
| FZ149 Bfa1-AID 1F | CCTATATGTATGAAATCAGGAACATGGTAATCAATTCGACAAAAGAT CGTACGCTGCAGGTCGAC | C-terminal AID tag for Bfa1 (forward) |
| FZ150 Bfa1-AID 1R | CTCAAGATAACGGTAAAGAAACAGTTATAAGAAGGCTAAAGGG ATCGATGAATTCGAGCTCG | C-terminal AID tag for Bfa1 (reverse) |
| FZ152 Bfa1 2F | GATGTTTTCGGAGACAATTTGGTTACTG | Genomic deletion Bfa1 (forward) |
| FZ153 Bfa1 2R | GACGCGAAATGTCGGCG | Genomic deletion Bfa1 (reverse) |
| GM474 | CGGGCCGTTTAGCGGAATGGATGAGTAAGTATGGTTGCACGATCTAAATAAATTCGTTTTCAATGATTAAAATAGCATAGTCGGGTTTTTCTTTTAGTTTCAGCTTTCCGCAACAGTATAATTTTATAAACCCTGGTTTTGGTTTTGTAGAGTGGTTTTGTGAACAACTGGTTTGTTGAAAAAGATCACTGGAATTA | Donor sequence template to insert an HO-cut site at *LYS2*. Use with plasmids bG059 or bG060. Red is the insertion for the HO-cut site. |
| FZ178 Lys2 gRNA 1 | ACCCATTTAACACCTGCCAT | gRNA for *LYS2*. Inserted into plasmid bRA90. |
| FZ179 Lys2 gRNA 2 | GGTTTGGCCGAAGGTTATAG | gRNA for *LYS2*. Inserted into plasmid bRA90. |
| FZ180 Rad9 1F | CGTACGCTGCAGGTCGAC | Forward primer to add AID tag to Rad9 in plasmid pFL36.1. Use with primer FZ181 and plasmid pJH2892. |
| FZ181 Rad91R | GGTGGTggcgcgccTTTTTAGCTAGT | Forward primer to add AID tag to Rad9 in plasmid pFL36.1. Use with primer FZ180 and plasmid pJH2892. Lower case is the location of a restriction enzyme site AscI. Red is a random sequence to assist with cutting by AscI. |
| FZ152 Bfa1 2F | GATGTTTTCGGAGACAATTTGGTTACTG | FW primer to check for Bfa1 deletion. 326bp upstream of start codon. Use with yeast haploid deletion collection. |
| FZ153 Bfa1 2R | GACGCGAAATGTCGGCG | Rev primer to check for Bfa1 deletion. 101bp downstream of stop codon. Use with yeast haploid deletion collection. |
| HTA1 gRNA1F | AACGTTACCATTGCCCAAGGgtttt | Guide RNA to cut HTA1 using CRISPR/Cas9. |
| HTA2 gRNA 1F | AATGTTACCATCGCCCAAGGgtttt | Guide RNA to cut HTA2 using CRISPR/Cas9. |
| HTB1 T129 gRNA | AGTACTCTTCCTCTACTCAAgatca | Guide RNA to cut HTB1 using CRISPR/Cas9. |
| HTB2 T129 gRNA | AATACTCCTCCTCTACTCAAgatca | Guide RNA to cut HTB2 using CRISPR/Cas9. |
| HTB1 T129A 80 mer | CTCTGAAGGTACTAGAGCTGTTACgAAGTACTCgTCCTCTGCTCAAGCATAATGAAATCACTTCCTTTGGTTATAATTAATAT | Repair template to replace threonine at 129 on HTB1 with alanine. |
| HTB1 T129E 80 mer | CTCTGAAGGTACTAGAGCTGTTACgAAGTACTCgTCCTCTGAACAAGCATAATGAAATCACTTCCTTTGGTTATAATTAATAT | Repair template to replace threonine at 129 on HTB1 with glutamic acid. |
| HTB2 T129E 80 mer | CTCCGAAGGTACTAGGGCTGTTACgAAATACTCgTCCTCTGAACAAGCCTAAGTCACTCACTAGGTATTGTGATTTAGTCATG | Repair template to replace threonine at 129 on HTB2 with glutamic acid. |
| HTB2 T129A 80 mer | CTCCGAAGGTACTAGGGCTGTTACgAAATACTCgTCCTCTGCTCAAGCCTAAGTCACTCACTAGGTATTGTGATTTAGTCATG | Repair template to replace threonine at 129 on HTB2 with alanine. |
| HTA1 S129A 80mer | AAGGTGGTGTTTTGCCAAACATCCATCAAAACTTGTTGCCAAAGAAGTCTGCCAAGGCTACCAAGGCTgctCAAGAATTA | Repair template to replace serine at 129 on HTA1 with alanine. |
| HTA1 S129E 80mer | AAGGTGGTGTTTTGCCAAACATCCATCAAAACTTGTTGCCAAAGAAGTCTGCCAAGGCTACCAAGGCTgaaCAAGAATTA | Repair template to replace serine at 129 on HTA1 with glutamic acid |
| HTA2 S129A 80mer | CCAAGGTGGTGTTTTGCCAAACATTCACCAAAACTTGTTGCCAAAGAAGTCTGCCAAGACTGCCAAAGCTgctCAAGAAC | Repair template to replace serine at 129 on HTA2 with alanine. |
| HTA2 S129E 80mer | CCAAGGTGGTGTTTTGCCAAACATTCACCAAAACTTGTTGCCAAAGAAGTCTGCCAAGACTGCCAAAGCTgaaCAAGAAC | Repair template to replace serine at 129 on HTA2 with glutamic acid. |
